# The Heidelberg Functional Foot Model—Application to Cavovarus and Equinovarus Feet

**DOI:** 10.1002/jfa2.70085

**Published:** 2025-09-15

**Authors:** Sarah Campos, Firooz Salami, Qiuyue Chen, Cornelia Putz, Stefanos Tsitlakidis, Sebastian I. Wolf

**Affiliations:** ^1^ Department for Orthopedics Heidelberg University Hospital Heidelberg Germany

**Keywords:** cavovarus, equinovarus, functional joint center calibration, multi‐segment foot model

## Abstract

Multisegment foot models have become increasingly important in biomechanical research and clinical gait analysis but often face limitations in defining joint positions. Often, they rely on simplified methods, such as using the midpoint between two markers to represent a joint, which lacks functional verification. In contrast, phenomenological angles, such as the medial arch angle, bypass joint center calculations, and offer sensitive, radiologically aligned indicators of foot mechanics. The Heidelberg functional foot model (HFFM) integrates functionally verified joint positions in combination with clinically relevant phenomenological measures, thereby enhancing clinical interpretability in gait analysis. The marker placement of the HFFM is based on the Heidelberg foot measurement method (HFMM). A four‐segment model (shank, hindfoot, forefoot, and hallux) is defined. Anatomical coordinate systems are established via regression formulas derived from functional joint parameter determination. Kinematic angles are compared with radiological measures. Additionally, six clinically relevant angles of the HFMM are integrated into the HFFM. The method is applied to cavovarus (CV, 19 feet), equinovarus (EV, 31 feet), and typically developed feet (TD, 88 feet). EV feet show more pronounced hindfoot varus and forefoot adduction than CV and TD feet. Within the parameters adopted from the HFMM, EV feet exhibit increased subtalar inversion and a stronger medial arch than CV. Significant correlations are identified between hindfoot/shank flexion, forefoot/hindfoot flexion and medial arch, and radiological angles. The HFFM is sensitive for analyzing equinvarus and cavovarus deformities without applying static offsets due to the functional approach. It enables calculating kinetics to better understand the biomechanics of foot deformities.

## 1 Introduction

A large number of multisegment foot models (MFMs) can be identified in the literature, differing in the number of markers and segments, definitions of segment coordinate systems (CS), and the joint centers between the segments depending on the clinical focus and application [1]. In clinical context, the Oxford Foot Model (OFM) is probably the most frequently used and consists of three segments (forefoot, hindfoot, and hallux) plus the shank segment [2, 3]. Among others [4, 5, 6], the OFM quantifies foot kinematics by calculating the movement between segments via Euler–Cardan angles following ISB recommendations [7]. However, in many approaches, the joint positions articulating foot segments are either not explicitly defined, as is the case in the OFM and the Milwaukee Foot Model [8], or these positions are derived via a simple midpoint approach, defining joint locations by the midpoint between two anatomically oriented foot markers as done, for example, in the Rizzoli model [5, 9], the Heidelberg foot measurement method (HFMM) [10], or more recently in the Amsterdam foot model [6]. The joint between forefoot and hindfoot, that is, the midfoot joint in some approaches, is defined by the midpoint of the markers on the navicular and the cuboid [11, 12] or the tuberosity of the metatarsal V [5] or including also a third marker on the tuberosity of the metatarsal I [10]. However, Bruening et al. pointed out that these ad hoc definitions are lacking functional verification [11].

In order to cope with the complexity of the foot anatomy, some methods do not exclusively use a rigid segment approach for quantifying foot kinematics in gait [6, 8, 13, 14, 15]. Following two‐dimensional angular quantifications, for example, from x‐ray imaging and potentially spanning across several bones within the foot, phenomenological parameters are described similar to joint angles. One example is the medial (longitudinal) arch. In the HFMM, the medial longitudinal arch is defined as the apex angle formed by the triangle of markers placed medially at the calcaneus, at the navicular, and at the head of the first metatarsal, respectively, and is most proximate to the measure “D1M‐Nav‐STL” in a recent work by Uhan et al. [15]. In many years of use, this quantity turned out to be a simple but sensitive measure for quantifying functional changes in foot deformities following surgical correction [16]. Recently, this phenomenological quantity has also been added to the OFM [15] since it is not covered by rigid segment kinematics. It appears that for clinical purposes 3D foot motion analysis should incorporate 3D kinematics obtained via a rigid segment approach as well as phenomenological parameters such as the medial arch.

Therefore, the aim of this work is to introduce a protocol for assessing 3D foot motion in gait which combines the typical approach of a rigid segment model (with hindfoot, forefoot, and hallux as done in many multisegment foot models) with the assessment of phenomenological parameters such as the medial arch of the HFMM. Further, the method should have a clear motivation for joint positions obtained from foot joint parameter estimation [17, 18, 19] which we therefore call “Heidelberg Functional Foot Model” (HFFM). With earlier findings in feet with planovalgus deformity [17], this protocol is applied to complementary deformities, that is, a cohort of patients presenting with cavovarus and equinovarus foot deformities. We hypothesize that the functional model is sensitive to foot deformities (here, equinovarus and cavovarus feet) and capable of finding key characteristics of these deformities. We also hypothesize that HFFM can produce foot kinematics without the need for post hoc applying anatomical offsets.

## 2 Materials and Methods

### 2.1 Marker Placement

**FIGURE 1:**
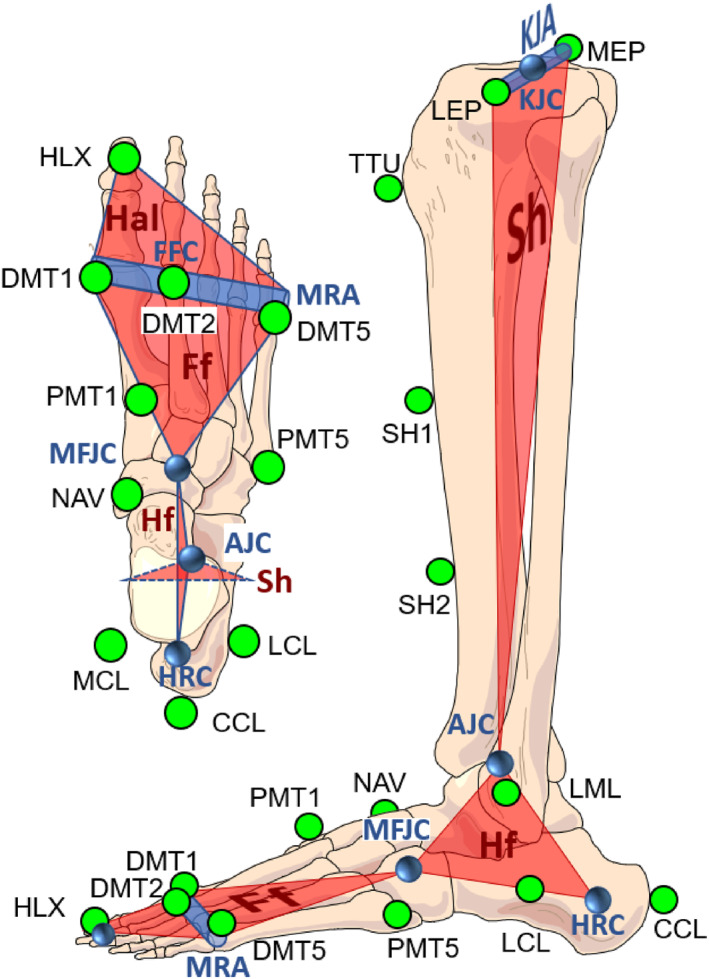
*Joints and rotation centers*: Knee joint axis (KJA) and knee joint center (KJC); ankle joint center (AJC); heel rotation center (HRC); midfoot joint center (MFJC); metatarsal rotation axis (MRA); *Segments*: shank (Sh); hindfoot (Hf); forefoot (Ff); and hallux (Hal); *Marker placement*: LEP, MEP: lat./med. epicondyle (cropped in the image); TTU: tibial tuberosity; SH1/2: two points at one‐third and two‐thirds along the medial side of the shin; LML, MML: lat./med. Malleoli; LCL, CCL, MCL: lat./dors./med. calcaneus; NAV: navicular; DMT1, PMT1: prox./dist. end first metatarsal; HLX: hallux; DMT2: dist. end second metatarsal; and DMT5, PMT5: dist./prox. end fifth metatarsal.

The marker set of the HFFM is identical to the protocol published in 2006 [10] which has proven to be reliable in several studies monitoring foot dysfunction and treatment [16, 20, 21, 22, 23, 24]. The only difference is the specific placement of the medial and lateral calcaneus marker (marker placement see Figure 1). Instead of the original device described in [10], a new hindfoot alignment device has been developed allowing for an improved reliability in marker placement [25].

### 2.2 Heidelberg Functional Foot Model Construct

In recent years, our group made progress in estimating the position of joint centers and reference points within the foot via the performance of specific active movement tasks. A circular motion of the unloaded foot in single limb stance may serve for determining the location of the ankle joint complex independent from bony landmarks [26]. As it turns out, this task provokes also substantial movement within the foot for determining the location of a “midfoot joint” which has no specific anatomic reference but which approximates the center of angular motion between hindfoot and forefoot on the basis of a two‐segment approach for the foot [17, 19]. Following the rocker concept of Perry [27], we also estimated a heel rotation center within the calcaneus and a metatarsal rotation axis within the forefoot [18]. This allows for constructing a functional foot model to be described here in further detail.

Typically, rigid segment modeling starts with defining technical coordinate systems which then are referenced to anatomical coordinate systems before joint kinematics is determined [28]. Here, we start with the functional determination of joint parameters (joint locations and orientiations). Key reference points are the knee joint center (KJC), the ankle joint center (AJC), the heel rotation center (HRC), the midfoot joint center (MFJC), and the forefoot center (FFC), respectively. Further, the orientation of the knee joint axis (KJA) is used to define the segment of the shank (Sh), whereas the orientation of the metatarsal rotation axis (MRA) is used to define the segment of the forefoot (Ff), respectively. The model as well as the marker set are visualized in Figure 1.

For calibrating joint parameters, the relative movement between foot markers in gait or in dedicated calibration movements is used. Namely, the positions of the AJC and the MFJC are derived from a specific movement task: During single‐limb stance, the subject is asked to perform a repetitive circular movement of the foot and ankle by drawing a circle with the hallux in the air as large as possible. In typical feet, this provokes substantial movement in the midfoot region as well as in the ankle as shown in earlier work [19]. In a similar manner, also the locations of the HRC and the MRA within the foot can be estimated via monitoring the markers on the foot when it is in contact to the ground in first and third rocker of gait, respectively [18]. In these studies, it turned out that typically developing subjects performed functional calibration with ease, resulting in highly repeatable results. However, in case of functional handicaps, the performance frequently was too poor to allow for functional joint center calibration. Hence, regression formulas were derived to obtain similar results also in cases where no direct functional calibration is possible. For the sake of robustness and feasibility and also for a more generalized approach, in this work, all reference points within the foot are derived directly from marker positions via regression formulas outlined below.

### 2.3 Joint Positions and Axes

The knee joint axis (KJA) is conventionally determined via markers on medial and lateral femoral epicondyles (MEP and LEP, respectively, see Figure 1) with the knee joint center (KJC) being the midpoint between these two markers. Similarly, for simplicity and also for better comparison with other approaches, the ankle joint center (AJC) is determined as the midpoint between the medial and lateral malleolus markers (MML and LML), since the effective joint center is located only slightly more anterior to this midpoint [26].

Similar to joints between body segments, the HRC is the pivotal point describing the center of rotational movement of the heel relative to the ground after heel strike which Perry introduced as “first rocker” or “heel rocker” in gait [27]. This functional point within the foot may also be determined via functional calibration as we showed earlier [18]. Since the HRC is located substantially posterior to the midpoint between the medial and lateral heel markers (MCL and LCL) [18], we decided to include also the dorsal heel marker (CCL; in conventional gait analysis simply referred to as “heel marker”) to establish a regression formula for the location of the HRC (see Table 1) based on the data of our earlier work [18].

**TABLE 1:** Definitions of the joint axes/center and the segments shank, hindfoot, forefoot, and hallux.

Joint axis/center	Determination	Implementation in HFFM
Knee joint axis (KJA)	Via anatomical landmarks	MEP to LEP
Knee joint center (KJC)	Via anatomical landmarks	Midpoint [LEP and MEP]
Ankle joint center (AJC)	Via anatomical landmarks	Midpoint [LML and MML]
Heel rotation center (HRC)	Via placement device HiAD	HRC(X) = 0.54CCL(X)−0.15LCL(X)+0.15MCL(X)
		HRC(Y) = 0.28CCL(Y)+0.43LCL(Y)+0.42MCL(Y)
		HRC(Z) = 0.29CCL(Z)+0.025LCL(Z)+1.74MCL(Z)
Midfoot joint center (MFJC)	Via anatomical landmarks	MFJC(X) = 0.16NAV(X)+0.25PMT1(X)+0.30PMT5(X)
		MFJC(Y) = 0.52NAV(Y)−0.17PMT1(Y)+0.26PMT5(Y)
		MFJC(Z) = 0.5NAV(Z)+0.5PMT5(Z)
Metatarsal rotation axis (MRA)	Via anatomical landmarks	(DMT1‐11 mm) to (DMT5‐11 mm)^a^
Forefoot center (FFC)	Via anatomical landmarks	FFC: Projection of DMT2 onto MRA
Segments		
Shank (Sh)	Origin	KJC
	anterior–posterior axis (x)	Cross product of Y‐axis and Z‐axis
	mediolateral axis (y)	KJC to LEP
	vertical axis (z)	KJC to AJC
Hindfoot (Hf)	Origin	HRC
	anterior–posterior axis (x)	HRC to MFJC
	mediolateral axis (y)	Cross product of the X‐axis and axis HRC to AJC
	vertical axis (z)	Cross product of the X‐ and Y‐axes
Forefoot (Ff)	Origin	MFJC
	anterior–posterior axis (x)	MFJC to FFC
	mediolateral axis (y)	Cross product of the X‐ and Z‐axes
	vertical axis (z)	Cross product of the X‐axis and MRA
Hallux (Hal)	Origin	FFC
	anterior–posterior axis (x)	FFC to HLX

*Note:* All the markers were converted to the local coordinate system of the foot according to refs. [15, 17].

^a^ Downward shift of 8 mm accounting for metatarsal head radius [29] plus a compensation for the marker radius of 3 mm.

Similar to the AJC also the midfoot joint center (MFJC) may be determined via functional calibration as we described earlier [17, 19]. According to this work, the midpoint between NAV and PMT5 is typically very close to the MFJC. Nevertheless, in order to improve the accuracy, here we decided to use a regression solution based on the marker locations of NAV, PMT1, and PMT5, respectively (see Table 1) which was proven to work equally well in children and adults and independent of the foot type, respectively [17].

The metatarsal rotation axis (MRA) may be determined via functional calibration as the “third rocker” or “forefoot rocker” [27] as we showed in the paper referenced above [18]. However, since the numerical solution in this case is not as stable as for the calibration of the other joints, we decided for an ad hoc solution to estimate the MRA to be in parallel to the axis between the markers on the base of first and fifth metatarsal (DMT1 and DMT5) but shifted downwards by 11 mm where 8 mm account for metatarsal head radius following results of Madjarevic et al. based on x‐ray imaging [29] with an additional compensation for the marker radius of 3 mm. Accordingly, the forefoot center (FFC) is defined as the projection of the DMT2 onto the MRA.

### 2.4 Segments

The shank segment (Sh) is defined by the location and direction of the KJA and the position of the AJC, respectively (see details in Table 1). For defining the hindfoot (Hf), the positions of the AJC, the MFJC, and the HRC are used, respectively. Similar to the shank, the forefoot (Ff) is defined via the center of a ball joint (here the MFJC) and the location and direction of a joint axis (here the MRA). The segment for the hallux is not modeled as a full 3D segment since only one marker (HLX) is applied.

### 2.5 Joint Kinematics

Euler–Cardan angles were calculated between shank and hindfoot (hindfoot flexion, varus, and rotation), between hindfoot and forefoot (forefoot flexion, supination, and adduction), and directly between shank and forefoot (forefoot flexion, supination, and adduction), respectively. The sequence of rotations, XYZ (sagittal, frontal, transverse), were performed following Grood and Suntay as well as the ISB recommendations [7, 30, 31] and identical to the Oxford Foot Model. Next to hallux flexion and abduction, the more phenomenological angles medial arch, medial arch inclination, subtalar eversion, and metatarsal I–V angle where calculated following the earlier Heidelberg foot measurement method [10].

### 2.6 Subjects and Protocol

Over a period of 2 years, patients were recruited for gait analysis with the inclusion criterion that they have presented in our outpatient clinic and have been diagnosed for a cavus foot deformity. In total, 33 patients (11 female) with varying underlying pathology participated (9 patients with hereditary motor sensory neuropathy, 7 patients with cerebral palsy, and 12 patients with unknown pathology). Based on kinematics results, all feet were post hoc classified into the subtypes of equinovarus (EV) and cavovarus (CV). EV feet were defined as having a positive first derivative (slope) of hindfoot/shank flexion at initial contact. This indicates a forefoot (first) contact and hence a functional equinus foot. The strength of the dorsiflexors and ankle ROM was assessed as part of a clinical examination to include only those EV feet with structural calf tightness or contractures. Thus, drop feet, which also may be classified as equinus deformities but presenting with a different kinematic pattern due to ankle dorsiflexor weakness (here defined as less than two in MRC scale), were excluded. Subsequently, data of 19 CV feet (11 subjects and age: 32.9 ± 16.2 years) and 31 EV feet (23 subjects age: 17.3 ± 10.8 years) were examined.

Additionally, data of 44 typically developing subjects (TD; age 25.5 ± 11.1 years, 25 female, and 88 feet) were included for reference. In the recruitment process, they underwent clinical examination including visual inspection of their feet. Reference subjects were only included when they had no clinical symptoms and showed no obvious foot deformities such as, for example, a nonpathological flat foot. The protocol had been approved by the institutional ethic committee (S‐850/2019) and each participant had provided informed written consent.

Examiners with more than three years of everyday experience applied 17 retro‐reflective markers on each foot in sitting position as referenced in the methods section. One static trial and at least 10 walking trials on a seven m walk way at a self‐selected speed were captured of each subject with a 12 camera Vero2.2 system (Vicon Motion Systems Ltd. Yarnton, Oxfordshire, UK).

Since the presented method is used for assessing foot deformities in clinical routine together with x‐ray imaging, we also compared three kinematic foot parameters (medial arch, hindfoot/shank flexion, and forefoot/hindfoot flexion) in static stance with sagittal weight‐bearing radiographic angles (if x‐rays were available) to check for similarities and deviations. Namely, calcaneal pitch (angle between plantar surface of the calcaneus and the weightbearing surface) [32, 33], metatarsal declination angle (angle between long axis of the first metatarsal and the weightbearing surface) [33], and Meary angle (angle between long axis of the talus and the long axis of the first metatarsal) were examined [32, 33]. In addition, these radiographic angles and the ankle ROM according to the Silferskiold test were used to characterize the severity of the deformity.

Postprocessing of the kinematic data were performed using MATLAB (MathWorks, Natick, MA, USA) and the program MoMo (MotionModeller) introduced by Simon et al. [10]. All walking trials were normalized and filtered using VCM (Vicon Clinical Manager) spline filter. Mean, standard deviation, maximum, and ROM were calculated for the CV, EV, and TD cohort. Statistical analysis was performed with IBM SPSS (Statistics for Windows, Armonk, NY). A single‐factor analysis of variance was performed to calculate significant kinematic differences in the mean, maxima, and ROM target values. The significance level was set at 5%. Bonferroni correction was applied for testing mean, max, and ROM of each joint angle but not for testing several joint angles independently. The Shapiro–Wilk test and bar charts with normal distribution curves were used to check the normal distribution. Although not all data showed a normal distribution, the analysis of variance was chosen to examine the differences.

## 3 Results

### 3.1 Foot Kinematics

**FIGURE 2:**
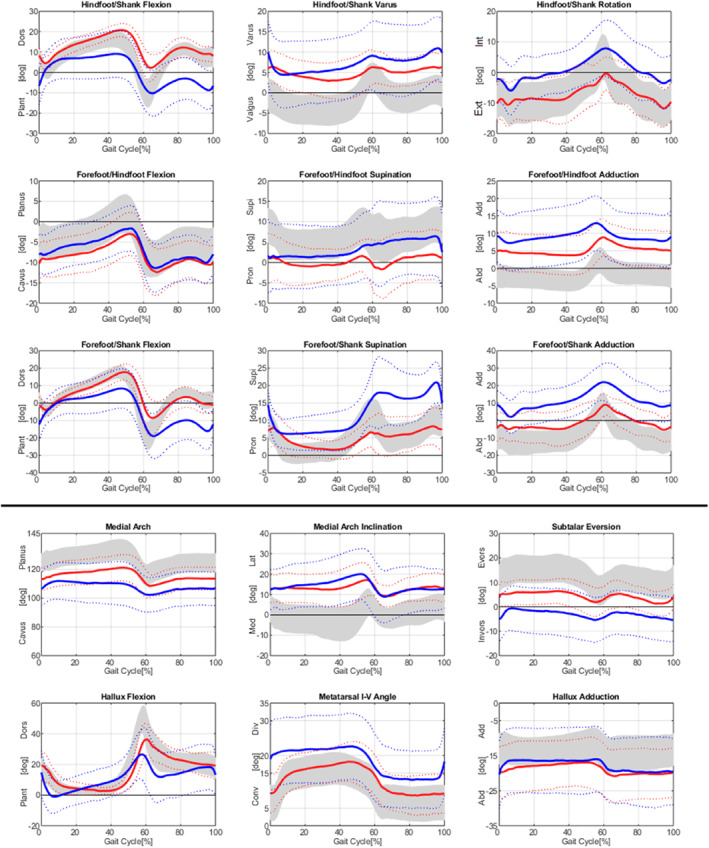
Foot kinematics of cavovarus (CV) feet (red), equinovarus (EV) feet (blue), and typical developed (TD) feet (gray) ± SD (dotted lines) in sagittal (first column), frontal (second column), and transverse plane (third column). The first 3 rows represent the kinematics of the (new) Heidelberg functional foot model (HFFM), the lower 2 rows represent angles according to the HFMM [10].

Foot kinematics of CV, EV, and TD are represented in Figure 2 and the resulting mean, maximum, and ROM are shown in Table 2 for each parameter.

**TABLE 2:** Mean, maximum (Max), and range of motion (ROM) with standard deviations (SDs) in the complete gait cycle for the groups cavovarus (CV), equinovarus (EV), and typical developed (TD).

*[Units in degrees]*	CV			EV			TD		
	Mean (SD)	Max (SD)	ROM (SD)	Mean (SD)	Max (SD)	ROM (SD)	Mean (SD)	Max (SD)	ROM (SD)
Hindfoot/Shank flexion	11.9 ± 2.5*	21.3 ± 3.2*	21.1 ± 6.0*	0.8 ± 9.7*	9.8 ± 10.7*	22.6 ± 5.9*	7.9 ± 3.1	16.9 ± 3.5	28.4 ± 5.5
Hindfoot/Shank varus	4.8 ± 3.6*	8.2 ± 3.8*	6.1 ± 2.0*	7.1 ± 7.6*	12.9 ± 8.2*	9.9 ± 3.8	−1.5 ± 3.4	4.5 ± 3.5	9.3 ± 2.9
Hindfoot/Shank rotation	−7.2 ± 6.0	2.4 ± 6.1	16.0 ± 4.2*	0.6 ± 7.0*	10.8 ± 8.8*	18.6 ± 6.1	−6.6 ± 5.8	6.5 ± 7.2	20.3 ± 4.3
Forefoot/Hindfoot flexion	−8.3 ± 4.5*	−2.6 ± 5.2*	10.4 ± 3.6	−6.8 ± 4.8*	−1.2 ± 5.4*	11.7 ± 3.8	−4.3 ± 4.0	2.1 ± 4.5	12.4 ± 2.8
Forefoot/Hindfoot supination	0.2 ± 4.8*	4.3 ± 5.2*	8.1 ± 2.1	3.4 ± 7.7	8.9 ± 9.1	10.6 ± 5.1	6.3 ± 3.9	11.1 ± 4.5	8.5 ± 2.1
Forefoot/Hindfoot abduction	5.2 ± 5.1*	10.0 ± 4.7*	7.1 ± 2.1	9.1 ± 7.5*	14.3 ± 7.4*	8.3 ± 2.0*	−2.1 ± 3.0	2.7 ± 3.5	6.8 ± 1.8
Forefoot/Shank flexion	4.2 ± 3.3	18.4 ± 4.3	28.8 ± 8.2*	−4.3 ± 9.4*	8.9 ± 11.2*	30.1 ± 8.4*	3.8 ± 2.4	17.9 ± 3.3	38.2 ± 6.9
Forefoot/Shank supination	4.8 ± 3.3	10.6 ± 4.8*	10.7 ± 4*	11.8 ± 4.4*	24.6 ± 7.5*	20.2 ± 7.5*	5.2 ± 2.9	13.7 ± 4.3	13.9 ± 3.9
Forefoot/Shank abduction	−1.3 ± 6.9*	12.0 ± 6.2	20.7 ± 5.8*	11.2 ± 8.8*	25.5 ± 10.3*	25.0 ± 7.3	−8.4 ± 5.8	9.7 ± 7.2	25.7 ± 5.1
Medial arch	115.6 ± 7.9*	121.6 ± 9.0*	15.0 ± 5.0*	108.0 ± 12.4*	113.8 ± 13.0*	13.7 ± 6.1*	126.2 ± 6.2	133.7 ± 6.8	18.6 ± 4.7
Medial arch inclination	13.0 ± 6.0*	20.0 ± 7.0*	13.7 ± 5.2	14.2 ± 10.8*	22.3 ± 11.7*	16.4 ± 4.7	0.0 ± 6.2	8.3 ± 6.1	14.3 ± 4.7
Metatarsal I–V angle	13.3 ± 4.9	18.9 ± 5.5	11.1 ± 3.5	18.4 ± 8.8*	23.8 ± 9.3*	11.7 ± 3.3	11.1 ± 4.5	16.9 ± 4.5	11.8 ± 1.9
Hallux flexion	15.2 ± 3.1	42.5 ± 7.4*	40.8 ± 8.0*	11.4 ± 10.5*	33.2 ± 15.7*	37.2 ± 14.2*	16.2 ± 3.6	52.6 ± 8.4	51.6 ± 8.1
Subtalar eversion	4.4 ± 4.6*	8.3 ± 4.1*	8.7 ± 2.8*	−3.0 ± 8.8*	0.9 ± 9.0*	8.8 ± 3.2*	12.2 ± 5.4	16.8 ± 5.7	11.4 ± 3.0
Hallux abduction	−18.8 ± 6.8*	−16 ± 6.7*	6.1 ± 2.4	−17.7 ± 9.1	−14.2 ± 9.2	7.5 ± 2.8*	−13.5 ± 4.3	−10.7 ± 4.3	5.5 ± 2.2

*Note:* The first 9 parameters are determined following the new approach of the Heidelberg functional foot model and the following 6 parameters are determined following the Heidelberg foot measurement method (HFMM) [10]. Significant differences between the EV or CV group and the TD group are marked with an asterisk (*). Significant differences between the deformity groups are highlighted in gray under the CV group. Significance level: *p* < 0.05.

Differences between the deformities can be observed especially in hindfoot/shank flexion. Both the EV and CV groups differed significantly from the TD group (MW: 7.9 ± 3.1°) (*p* < 0.001). The EV cohort exhibits greater plantarflexion (mean: 0.8 ± 9.7°), whereas the CV cohort is more dorsiflexed (mean: 11.9 ± 2.5°) relative to TD, respectively. Although the ROM is approximately the same, it is less than in TD (CV: 21.6 ± 6.0°, EV: 22.6 ± 5.9°, and TD: 28.4 ± 5.5°). In frontal plane, EV feet have slightly more hindfoot varus (mean: 7.1 ± 7.6°) than CV feet (mean: 4.8 ± 3.6°). In hindfoot/shank rotation, the CV cohort does not deviate from TD, whereas the EV cohort shows slightly more internal rotation. Minimal differences in forefoot/hindfoot flexion are observed between CV and EV, with both cohorts tending toward a cavus deformity. In transverse plane, CV and EV deformities exhibit maximum forefoot adduction of 10.0 ± 4.7° and 14.3 ± 7.4°, respectively. Therefore, they are more adducted than TD feet (max: 2.7 ± 3.5°).

Regarding the parameters of the HFMM [10], the EV cohort demonstrates more pronounced subtalar eversion (mean: −3.0 ± 8.8°) and deviates significantly from the CV cohort (mean: 4.4 ± 4.6° and *p* = 0.001) as well as to the TD cohort (mean: 12.2 ± 5.4° and *p* < 0.001). The medial arch shows the largest difference, with more cavus in EV feet (mean: 108 ± 12.4°) compared to CV feet (mean: 115.6 ± 7.9°).

### 3.2 Radiographs and Clinical Examination

The summarized data on x‐rays and clinical examination are reported in Table 3. Radiographs were available in 44 out of 50 feet. The mean Meary angle and the metatarsal declination angle in the CV group (10 subjects and 15 feet) and the EV group (22 subjects and 29 feet) were almost identical (see Table 3). The calcaneal pitch was by 6.2° significantly larger in the CV group compared to the EV group.

**TABLE 3:** Data obtained by x‐ray and clinical examination.

	CV	EV
No. subjects	10	22
No. feet	15	29
x‐ray		
Meary angle	8.5 ± 4.5°	8.4 ± 4.8°
Calcaneal pitch	24.7 ± 5.5°	18.5 ± 5.0°
Metatarsal declination angle	30.1 ± 5.1°	31.6 ± 2.8°
Clinical examination		
DF in knee ext	2.4 ± 3.5°	1.4 ± 5.0°
DF in knee flex	11.5 ± 8.2°	3.8 ± 7.0°
Plantarflexion	42.4 ± 12.1°	42.9 ± 9.6°

A significant Pearson correlation was found between the medial arch (mean of CV: 119.9 ± 8.5° and EV: 111.8 ± 13.7°) and the Meary angle (*r* = −0.404 and *p* = 0.006) as well as with the metatarsal declination angle (*r* = −0.608 and *p* < 0.001), respectively. The metatarsal declination angle further correlated with hindfoot/shank Flexion (*r* = 0.401, *p* = 0.023, and mean of CV: 12.4 ± 3.5° and EV: 6.6 ± 11.3°) and forefoot/hindfoot flexion (*r* = −0.353, *p* = 0.047, and mean of CV: −11.6 ± 6.7° and EV: −9.7 ± 8.8°).

Passive plantarflexion and passive dorsiflexion of the ankle in knee extension were very similar between groups, whereas passive dorsiflexion in 90° knee flexion deviated by 7.7° with the CV group showing significantly larger dorsiflexion compared to the EV group (see Table 3).

## 4 Discussion

Summarizing the knowledge of our previous studies regarding functional joint parameter estimation, the aim of this study was to define a functionally motivated foot model, including a rigid‐segment approach, and to combine it with established phenomenological foot angles obtained via the HFMM. The method was applied to patients with CV and EV deformity, as well as to TD subjects, for comparison.

### 4.1 Methodology

According to the recommendations of the Gait and Clinical Movement Society (GCMAS) [34] and most of the published multisegment foot models [2, 3, 4, 10, 35], the HFFM subdivides the foot into four segments: shank, hindfoot, forefoot, and hallux. According to Schallig et al., using the midpoint of markers instead of individual markers already leads to more robust CS [6]. The joint centers, namely, HRC and MFJC, which are used for segment axis definition of the hindfoot, are calculated via regression equations that take the location of three markers into account, resulting in joint centers located within the foot. The determination of these joint centers is based on the movement of segments relative to each other. This approach allows for the definition of functionally based anatomical CS, eliminating the need for a static reference position. This reasoning is supported by the correlation of radiographic angles with the Medial Arch, Hindfoot/Shank slexion and forefoot/hindfoot flexion. High correlations were also found in Pes cavovarus for hindfoot/shank flexion using the Oxford Foot Model, but no relation to radiographic angles in the sense of a predictor was observed for forefoot/hindfoot flexion [36].

In this approach, the orientation of the hindfoot long axis (line from heel rotation center to midfoot joint center) in sagittal view corresponds well with the calcaneal pitch assessed in lateral weight‐bearing x‐ray images. Similarly, the forefoot long axis (line from midfoot joint center to metatarsal rotation axis) corresponds well with the metatarsal declination angle, respectively. Hence, there is a good correspondence between sagittal x‐ray imaging of the foot and the sagittal foot parameters obtained via this approach.

Furthermore, the lateral and medial calcaneus markers are reliably placed with the HiAD [37] despite the difficulties in identifying bony landmarks on the hindfoot [38]. These markers, along with the dorsal calcaneus marker, are used to determine the HRC, which serves as the origin of the hindfoot segment. This approach allows for the description of the clinically important varus or valgus alignment of the hindfoot relative to the shank. It has been demonstrated in the Oxford Foot Model that when defining a hindfoot axis with the dorsal calcaneus marker, the CS is highly sensitive to the placement of the marker [39].

The long and mediolateral axis of the forefoot is created by correcting the height of the markers on the metatarsal heads. Based on consistent results of a study on metatarsal bone thickness measurements [29] and previous investigations by our group in static stance on marker height (DMT1 and DMT5) in TD and deformed feet of children and adults, we shifted the original marker positions of DMT1 and DMT5 by the sum of marker radius (3 mm) and metatarsal head radius (8 mm). DMT2 is then projected onto this shifted axis, creating a virtual marker at the metatarsal heads, which serves as joint center.

It may appear that the presented methodology is just another foot model next to many existing ones in the literature. However, with this approach, the intuitive concept of foot rockers in the stance phase of gait as established by Perry [27] is transferred to a (simple) foot‐ground contact model. Ground contacts are defined by the heel rotation center and the metatarsal rotation axis. Together with the midfoot joint derived as functional junction between hindfoot and forefoot, inverse dynamics calculations become feasible. This methodological step is beyond the scope of this work and will be elaborated in future studies.

### 4.2 Cavus Foot Deformities

In this study, CV and EV deformities were monitored because of their high prevalence in cerebral palsy, hereditary motor sensory neuropathy (HMSN), and other neurological disorders [40] with still limited research [23, 38, 41, 42].

Cavus feet are associated with an increased calcaneal pitch (around 30°) [43, 44, 45, 46] whereas TD feet typically show a Meary angle of 0° and a calcaneal pitch of only 18° [33, 45]. With 8.5° in CV and 8.4° in EV feet, all subjects in our study showed a pronounced Meary angle in x‐ray imaging characterizing a forefoot‐driven cavus deformity. In contrast, the calcaneal pitch was observed to be increased only in CV feet with a mean angle of approximately 25°. Therefore, only the CV group demonstrated a hindfoot‐driven cavus and were on average more dorsiflexed than TD feet due to cavus deformity as described earlier [23, 38]. The foot kinematics showed more hindfoot plantarflexion in the EV group compared to the CV group due to forefoot first contact.

In the frontal plane, EV feet were slightly more in a varus position compared to CV feet as the equinus deformity increases the hindfoot varus position due to the altered weight bearing. This is in line with the finding of larger hindfoot inversion reported in ref. [47]. Similar to this work, we found a higher incidence of forefoot pronation in EV feet. The forefoot/hindfoot kinematics showed pronounced forefoot adduction in both groups compared to TD, with a greater increase in CV feet. These findings are consistent with results of CV feet in the Oxford Foot Model [36]. Furthermore, a rigid cavus deformity that may occur with persistent calf muscle contracture may be responsible for the reduced ROM in all parameters in CV feet [44].

To determine the extent of the cavus deformity in the CV and EV groups, the forefoot/hindfoot flexion and medial arch are both advantageous. The forefoot/hindfoot flexion includes all markers on the forefoot and hindfoot segments, thereby enabling a cavus deformity of the metatarsals I–V to be demonstrated. In contrast, the medial arch exclusively reflects the medial aspect of the deformity and therefore the plantarflexion of metatarsal I. Among the examined cohorts, EV feet showed a pronounced cavus assessed at the medial forefoot (metatarsal I) in contrast to CV feet, whereas the CV group had demonstrating with an increased cavus assessed across metatarsal I–V. Therefore, both parameters should be reported to analyze foot function.

## 5 Conclusion

In summary, the HFFM (1) makes use of phenomenological parameters of foot function [10], (2) adds a rigid two‐segment approach with functionally based anatomical coordinate systems which eliminates the need for a static reference position and offset correction, and (3) defines rotational joint centers derived from calibration movements allowing for calculation of joint kinetics in future work. In its application presented in this work, (4) it allows for sensitively detecting cavus foot deformities, and (5) allows for distinguishing if the cavus deformity is present primarily in the first ray (i.e., medial arch) or rather in all metatarsals I–V.

## Author Contributions

**Sarah Campos:** investigation, methodology, software, validation, visualization, writing original draft. **Firooz Salami:** data curation, formal analysis, investigation, methodology, software, supervision, validation, writing – review and editing. **Qiuyue Chen:** investigation, methodology, writing – review and editing. **Cornelia Putz:** formal analysis, investigation, supervision, writing – review and editing **Stefanos Tsitlakidis:** formal analysis, investigation, supervision, writing – review and editing. **Sebastian I. Wolf:** conceptualization, funding acquisition, investigation, methodology, project administration, resources, supervision, writing – review and editing.

## Conflicts of Interest

The authors declare no conflicts of interest.

## Data Availability

The data and the code for modeling of this work are available upon request to the first author.

## References

[1] A. Leardini , P. Caravaggi , T. Theologis , and J. Stebbins , “Multi‐Segment Foot Models and Their Use in Clinical Populations,” Gait & Posture 69 (2019): 50–59, 10.1016/j.gaitpost.2019.01.022. 30665039

[2] M. C. Carson , M. E. Harrington , N. Thompson , J. J. O’Connor , and T. N. Theologis , “Kinematic Analysis of a Multi‐Segment Foot Model for Research and Clinical Applications: A Repeatability Analysis,” Journal of Biomechanics 34, no. 10 (2001): 1299–1307, 10.1016/s0021-9290(01)00101-4. 11522309

[3] J. Stebbins , M. Harrington , N. Thompson , A. Zavatsky , and T. Theologis , “Repeatability of a Model for Measuring Multi‐Segment Foot Kinematics in Children,” Gait & Posture 23, no. 4 (2006): 401–410, 10.1016/j.gaitpost.2005.03.002. 15914005

[4] S. M. Kidder , F. S. Abuzzahab , G. F. Harris , and J. E. Johnson , “A System for the Analysis of Foot and Ankle Kinematics During Gait,” IEEE Transactions on Rehabilitation Engineering 4, no. 1 (1996): 25–32, 10.1109/86.486054. 8798069

[5] A. Leardini , M. G. Benedetti , L. Berti , D. Bettinelli , R. Nativo , and S. Giannini , “Rear‐Foot, Mid‐Foot and Fore‐Foot Motion During the Stance Phase of Gait,” Gait & Posture 25, no. 3 (2007): 453–462, 10.1016/j.gaitpost.2006.05.017. 16965916

[6] W. Schallig , J. C. van den Noort , M. Piening , et al., “The Amsterdam Foot Model: A Clinically Informed Multi‐Segment Foot Model Developed to Minimize Measurement Errors in Foot Kinematics,” Journal of Foot and Ankle Research 15, no. 1 (2022): 46, 10.1186/s13047-022-00543-6. 35668453 PMC9172122

[7] A. Leardini , J. Stebbins , H. Hillstrom , P. Caravaggi , K. Deschamps , and A. Arndt , “ISB Recommendations for Skin‐Marker‐Based Multi‐Segment Foot Kinematics,” Journal of Biomechanics 125 (2021): 110581, 10.1016/j.jbiomech.2021.110581. 34217032

[8] S. M. Kidder , F. S. Abuzzahab , G. F. Harris , and J. E. Johnson , “A System for the Analysis of Foot and Ankle Kinematics During Gait,” IEEE Transactions on Rehabilitation Engineering 4, no. 1 (1996): 25–32, 10.1109/86.486054. 8798069

[9] A. Leardini , M. G. Benedetti , F. Catani , L. Simoncini , and S. Giannini , “An Anatomically Based Protocol for the Description of Foot Segment Kinematics During Gait,” Clinical Biomechanics 14, no. 8 (1999): 528–536, 10.1016/s0268-0033(99)00008-x. 10521637

[10] J. Simon , L. Doederlein , A. S. McIntosh , D. Metaxiotis , H. G. Bock , and S. I. Wolf , “The Heidelberg Foot Measurement Method: Development, Description and Assessment,” Gait & Posture 23, no. 4 (2006): 411–424, 10.1016/j.gaitpost.2005.07.003. 16157483

[11] D. A. Bruening , K. M. Cooney , and F. L. Buczek , “Analysis of a Kinetic Multi‐Segment Foot Model. Part I: Model Repeatability and Kinematic Validity,” Gait & Posture 35, no. 4 (2012): 529–534, 10.1016/j.gaitpost.2011.10.363. 22421190

[12] C. J. Nester , A. M. Liu , E. Ward , et al., “In Vitro Study of Foot Kinematics Using a Dynamic Walking Cadaver Model,” Journal of Biomechanics 40, no. 9 (2007): 1927–1937, 10.1016/j.jbiomech.2006.09.008. 17081548

[13] M. E. Ness , J. Long , R. Marks , and G. Harris , “Foot and Ankle Kinematics in Patients With Posterior Tibial Tendon Dysfunction,” Gait & Posture 27, no. 2 (2008): 331–339, 10.1016/j.gaitpost.2007.04.014. 17583511

[14] P. Saraswat , B. A. MacWilliams , and R. B. Davis , “A Multi‐Segment Foot Model Based on Anatomically Registered Technical Coordinate Systems: Method Repeatability in Pediatric Feet,” Gait & Posture 35, no. 4 (2012): 547–555, 10.1016/j.gaitpost.2011.11.022. 22192872

[15] J. Uhan , A. Kothari , A. Zavatsky , and J. Stebbins , “Using Surface Markers to Describe the Kinematics of the Medial Longitudinal Arch,” Gait & Posture 102 (2023): 118–124, 10.1016/j.gaitpost.2023.03.016. 37003196

[16] T. Dreher , S. I. Wolf , D. Heitzmann , C. Fremd , M. C. Klotz , and W. Wenz , “Tibialis Posterior Tendon Transfer Corrects the Foot Drop Component of Cavovarus Foot Deformity in Charcot‐Marie‐Tooth disease,” Journal of Bone and Joint Surgery 96, no. 6 (2014): 456–462, 10.2106/jbjs.l.01749. 24647501

[17] S. Campos , F. Salami , M. Götze , K. Gather , and S. I. Wolf , “Using Functional Calibration Methods to Estimate the Midfoot Joint Center in Planovalgus Feet,” Journal of Biomechanics 180 (2025): 112493, 10.1016/j.jbiomech.2025.112493. 39793510

[18] F. Salami , S. Campos , A. R. Musagara , and S. I. Wolf , “Modeling Foot Rockers via Functional Calibration for Use in Clinical Gait Analysis,” Gait & Posture 111 (2024): 122–125, 10.1016/j.gaitpost.2024.04.022. 38678930

[19] F. Salami , M. Götze , S. Campos , J. Leboucher , S. Hagmann , and S. I. Wolf , “Estimation of a Midfoot Joint Center in Typically Developed Adults Using Functional Calibration Methods,” Gait & Posture 97 (2022): 203–209, 10.1016/j.gaitpost.2022.08.013. 35988436

[20] J. C. Bopple , M. Tanner , S. Campos , et al., “Short‐Term Results of Gait Analysis With the Heidelberg Foot Measurement Method and Functional Outcome After Operative Treatment of Ankle Fractures,” Journal of Foot and Ankle Research 15, no. 1 (2022): 2, 10.1186/s13047-021-00505-4. 34998420 PMC8742407

[21] L. P. Bartsch , M. Schwarze , J. Block , et al., “Hindfoot Flexibility Influences the Biomechanical Effects of Laterally Wedged Insoles and Ankle‐Foot Orthoses in Medial Knee Osteoarthritis,” Archives of Physical Medicine and Rehabilitation 103, no. 9 (2022): 1699–1706, 10.1016/j.apmr.2022.02.012. 35288097

[22] B. Kuni , J. Mussler , E. Kalkum , H. Schmitt , and S. I. Wolf , “Effect of Kinesiotaping, Non‐Elastic Taping and Bracing on Segmental Foot Kinematics During Drop Landing in Healthy Subjects and Subjects With Chronic Ankle Instability,” Physiotherapy 102, no. 3 (2016): 287–293, 10.1016/j.physio.2015.07.004. 26422550

[23] N. A. Beckmann , S. I. Wolf , D. Heitzmann , A. Wallroth , S. Müller , and T. Dreher , “Cavovarus Deformity in Charcot‐Marie‐Tooth Disease: Is There a Hindfoot Equinus Deformity That Needs Treatment?,” Journal of Foot and Ankle Research 8, no. 1 (2015): 65, 10.1186/s13047-015-0121-6. 26617675 PMC4661993

[24] B. Kuni , S. I. Wolf , F. Zeifang , and M. Thomsen , “Foot Kinematics in Walking on a Level Surface and on Stairs in Patients With Hallux Rigidus Before and After Cheilectomy,” Journal of Foot and Ankle Research 7, no. 1 (2014): 13, 10.1186/1757-1146-7-13. 24524773 PMC3925775

[25] S. Campos , F. Salami , M. Karrasch , A. R. Musagara , S. Hagmann , and S. I. Wolf , “A New Alignment Device for Standardization of Marker Placement on the Hindfoot,” Gait & Posture 104 (2023): 116–119, 10.1016/j.gaitpost.2023.06.008. 37379737

[26] F. Salami , S. I. Wolf , J. Simon , et al., “Estimation of Ankle Joint Parameters in Typically Developed Adults Using Functional Calibration Methods,” Gait & Posture 77 (2020): 95–99, 10.1016/j.gaitpost.2020.01.016. 32004952

[27] J. P. Perry , Gait Analysis Normal and Pathological Function (Slack, 1992).

[28] A. Cappozzo , U. Della Croce , A. Leardini , and L. Chiari , “Human Movement Analysis Using Stereophotogrammetry; Part 1: Theoretical Background,” Gait & Posture 21, no. 2 (2005): 186–196, 10.1016/j.gaitpost.2004.01.010. 15639398

[29] M. Madjarevic , R. Kolundzic , V. Trkulja , M. Mirkovic , and M. Pecina , “Biomechanical Analysis of Functional Adaptation of Metatarsal Bones in Statically Deformed Feet,” International Orthopaedics 33, no. 1 (2009): 157–163, 10.1007/s00264-008-0622-z. 18663446 PMC2899242

[30] E. S. Grood and W. J. Suntay , “A Joint Coordinate System for the Clinical Description of Three‐Dimensional Motions: Application to the Knee,” Journal of Biomechanical Engineering 105, no. 2 (1983): 136–144, 10.1115/1.3138397. 6865355

[31] G. Wu , S. Siegler , P. Allard , et al., “ISB Recommendation on Definitions of Joint Coordinate System of Various Joints for the Reporting of Human Joint Motion—Part I: Ankle, Hip, and Spine,” Journal of Biomechanics 35, no. 4 (2002): 543–548, 10.1016/s0021-9290(01)00222-6. 11934426

[32] J. R. Davids , T. W. Gibson , and L. I. Pugh , “Quantitative Segmental Analysis of Weightbearing Radiographs of the Foot and Ankle for Children: Normal Alignment,” Journal of Pediatric Orthopaedics 25, no. 6 (2005): 769–776, 10.1097/01.bpo.0000173244.74065.e4. 16294134

[33] B. M. Lamm , P. A. Stasko , M. G. Gesheff , and A. Bhave , “Normal Foot and Ankle Radiographic Angles, Measurements, and Reference Points,” Journal of Foot & Ankle Surgery 55, no. 5 (2016): 991–998, 10.1053/j.jfas.2016.05.005. 27320694

[34] K. Deschamps , F. Staes , P. Roosen , et al., “Body of Evidence Supporting the Clinical Use of 3D Multisegment Foot Models: A Systematic Review,” Gait & Posture 33, no. 3 (2011): 338–349, 10.1016/j.gaitpost.2010.12.018. 21251834

[35] A. E. Hunt , R. M. Smith , M. Torode , and A.‐M. Keenan , “Inter‐Segment Foot Motion and Ground Reaction Forces Over the Stance Phase of Walking,” Clinical biomechanics 16, no. 7 (2001): 592–600, 10.1016/s0268-0033(01)00040-7. 11470301

[36] H. Bohm , L. Döderlein , A. Fujak , and C. U. Dussa , “Is There a Correlation Between Static Radiographs and Dynamic Foot Function in Pediatric Foot Deformities?,” Foot and Ankle Surgery 26, no. 7 (2020): 801–809, 10.1016/j.fas.2019.10.006. 31694790

[37] S. Campos , et al., A New Alignment Device for Standardization of Marker Placement on the Hindfoot (Heidelberg University Hospital: Publikation in Vorbereitung, 2023): Clinic for Orthopedics and Trauma Surgery. 10.1016/j.gaitpost.2023.06.008 37379737

[38] K. M. Kruger , A. Graf , A. Flanagan , et al., “Segmental Foot and Ankle Kinematic Differences Between Rectus, Planus, and Cavus Foot Types,” Journal of Biomechanics 94 (2019): 180–186, 10.1016/j.jbiomech.2019.07.032. 31420153

[39] A. M. H. Paik , J. Stebbins , A. Kothari , and A. B. Zavatsky , “Effect of Marker Placement on Oxford Foot Model Hindfoot Segment Axes,” supplement, Journal of Foot and Ankle Research 7, no. S1 (2014): A62, 10.1186/1757-1146-7-s1-a62.

[40] P. A. O'Connell , L. D'Souza , S. Dudeney , and M. Stephens , “Foot Deformities in Children With Cerebral Palsy,” Journal of Pediatric Orthopaedics 18, no. 6 (1998): 743–747, 10.1097/01241398-199811000-00009. 9821129

[41] A. K. Buldt , P. Levinger , G. S. Murley , H. B. Menz , C. J. Nester , and K. B. Landorf , “Foot Posture Is Associated With Kinematics of the Foot During Gait: A Comparison of Normal, Planus and Cavus Feet,” Gait & Posture 42, no. 1 (2015): 42–48, 10.1016/j.gaitpost.2015.03.004. 25819716

[42] W. Schallig , M. Piening , L. Quirijnen , M. M. Witbreuk , A. I. Buizer , and M. M. van der Krogt , “Multi‐Segment Foot Kinematics During Gait in Children With Spastic Cerebral Palsy,” Gait & Posture 110 (2024): 144–149. 38608379 10.1016/j.gaitpost.2024.03.014

[43] J. Berciano , E. Gallardo , A. García , A. L. Pelayo‐Negro , J. Infante , and O. Combarros , “New Insights Into the Pathophysiology of Pes Cavus in Charcot–Marie–Tooth Disease Type 1A Duplication,” Journal of Neurology 258, no. 9 (2011): 1594–1602, 10.1007/s00415-011-6094-x. 21590514

[44] C. Maynou , C. Szymanski , and A. Thiounn , “The Adult Cavus Foot,” EFORT Open Reviews 2, no. 5 (2017): 221–229, 10.1302/2058-5241.2.160077. 28630759 PMC5467681

[45] A. J. Rosenbaum , J. Lisella , N. Patel , and N. Phillips , “The Cavus Foot,” Medical Clinics 98, no. 2 (2014): 301–312, 10.1016/j.mcna.2013.10.008. 24559876

[46] R. L. Samilson and W. Dillin , “Cavus, Cavovarus, and Calcaneocavus an Update,” Clinical Orthopaedics and Related Research 177 (1983): 125–132, 10.1097/00003086-198307000-00019. 6861385

[47] G. de Vries , K. Roy , and V. Chester , “Using Three‐Dimensional Gait Data for Foot/Ankle Orthopaedic Surgery,” Open Orthopaedics Journal 3, no. 1 (2009): 89–95, 10.2174/1874325000903010089. 19997521 PMC2788742

